# Patient-Level Multidimensional Response Phenotypes in Obesity-Associated Type 2 Diabetes: A 12-Month Real-World Cohort Study

**DOI:** 10.3390/jcm15145671

**Published:** 2026-07-20

**Authors:** Ioana Bujdei-Tebeică, Anca Mihaela Pantea-Stoian, Doina Andrada Mihai, Simona Diana Ștefan, Cristian Serafinceanu

**Affiliations:** 1 Department of Diabetes, Nutrition and Metabolic Diseases, Carol Davila University of Medicine and Pharmacy, 050474 Bucharest, Romania; 2 National Institute of Diabetes, Nutrition and Metabolic Diseases, 020475 Bucharest, Romania

**Keywords:** type 2 diabetes mellitus, obesity-associated diabetes, responder phenotypes, multidimensional response, response heterogeneity, HbA1c, fat mass, high-sensitivity C-reactive protein, handgrip strength, real-world evidence

## Abstract

**Background/Objectives**: Type 2 diabetes mellitus (T2DM) has been evaluated through a glucocentric model centered on hyperglycemia, glycated haemoglobin (HbA1c) targets, and complications. However, obesity-associated T2DM reflects a broader metabolic ecosystem in which glycemic control interacts with adiposity, inflammation, muscle function, renal stability, and treatment mechanisms. Therapeutic response may be better captured through patient-level responder phenotypes than through mean changes in isolated outcomes. We aimed to determine, in adults with obesity-associated T2DM maintained on stable 12-month metformin-based regimens, the distribution of concordant versus discordant glycemic–adiposity response phenotypes and the frequency of high multidimensional response, and to describe how these phenotypes were distributed across treatment classes. **Methods**: This secondary patient-level analysis used a real-world cohort from a Bucharest tertiary diabetes center. Of 291 screened adults, 166 completed 12-month follow-up without treatment change. Regimens included metformin alone or combined with sulfonylurea, DPP-4 inhibitor, SGLT2 inhibitor, GLP-1 receptor agonist, or insulin. Responses were defined as HbA1c reduction ≥ 0.5 percentage points, fat mass reduction ≥ 5%, hs-CRP reduction ≥ 20%, and handgrip strength decline ≤ 1 kg. Primary phenotypes and a 0–4 multidimensional score were derived. **Results**: Mean age was 59.7 ± 13.0 years, BMI 36.3 ± 4.4 kg/m^2^, and baseline HbA1c 8.9 ± 1.0%. Glycemic–adiposity, glycemic-only, adiposity-only, and low/non-response phenotypes occurred in 49 (29.5%), 88 (53.0%), 9 (5.4%), and 20 (12.0%) patients, respectively. A high multidimensional response occurred in 78 (47.0%), and complete response in 25 (15.1%). GLP-1 receptor agonists showed the most consistent concordant glycemic–adiposity response, whereas insulin was predominantly glycemic-only. **Conclusions**: In obesity-associated T2DM, therapeutic response was heterogeneous and frequently discordant across metabolic domains. Patient-level responder phenotyping may complement conventional HbA1c-centered evaluation by identifying response quality across glycemic, adiposity, inflammatory, and functional dimensions. This approach may support more individualized diagnostic interpretation and treatment optimization in real-world diabetes care.

## 1. Introduction

For several decades, the clinical understanding of type 2 diabetes mellitus (T2DM) was dominated by a primarily glucocentric model. In this framework, hyperglycemia represented the central diagnostic abnormality, glycated hemoglobin was the main therapeutic target, and treatment success was largely defined by the degree of glucose lowering [1]. This approach had a justified clinical rationale, namely, chronic hyperglycemia is directly linked to microvascular complications, and sustained glycemic control reduces the risk of retinopathy, nephropathy, neuropathy, and other diabetes-related outcomes [2,3]. Consequently, the traditional management of T2DM focused on achieving glycemic targets and treating complications once they emerged [1,2,3].

However, this model has progressively expanded. T2DM is now understood not only as a disorder of glucose regulation, but also as a complex cardio-reno-metabolic disease in which insulin resistance, adiposity, beta-cell dysfunction, ectopic fat accumulation, chronic low-grade inflammation, endothelial dysfunction, renal impairment, and functional decline interact over time [4,5]. In many patients, hyperglycemia is only one visible component of a broader metabolic disturbance. This is especially evident in obesity-associated T2DM, where excess adipose tissue contributes to insulin resistance, systemic inflammation, altered lipid metabolism, and increased cardiovascular risk [6,7]. Therefore, a therapeutic strategy that improves HbA1c without addressing adiposity, inflammation, or functional vulnerability may be incomplete from a biological and clinical perspective [4,6].

This broader perspective has changed the way antidiabetic therapies are evaluated. Earlier treatment paradigms mainly compared glucose-lowering potency, whereas current approaches increasingly consider body weight, fat mass, cardiovascular protection, renal outcomes, hypoglycemia risk, hepatic steatosis, inflammation, and patient-centered functional outcomes [8]. The emergence of glucagon-like peptide-1 receptor agonists and sodium-glucose cotransporter-2 inhibitors has accelerated this shift, because these agents exert clinically relevant effects beyond HbA1c reduction [9]. In contrast, other therapeutic strategies, such as insulin intensification or sulfonylurea treatment, may improve glycemia while having less favorable or neutral effects on weight and adiposity [10]. These divergent profiles illustrate why mean HbA1c reduction alone can no longer fully describe therapeutic benefit in T2DM.

A further limitation of conventional treatment evaluation is that group-level averages may obscure substantial patient-level heterogeneity. In routine clinical practice, patients do not respond uniformly to the same intervention. Some achieve meaningful glycemic improvement without relevant weight or fat mass reduction; others lose weight or adiposity without major glycemic benefit; some improve across several domains simultaneously; and others show limited response despite treatment intensification [11]. These discordant response patterns are clinically important because they suggest that therapeutic success is not a single-dimensional phenomenon. Rather, response to antidiabetic therapy may represent a composite biological pattern emerging from the interaction between treatment mechanism, baseline phenotype, diabetes duration, adiposity, inflammation, renal function, muscle reserve, adherence, nutrition, and physical activity [12].

Previous work on response heterogeneity to GLP-1 receptor agonists has highlighted this issue by showing that reductions in HbA1c and body weight do not necessarily occur together in the same patient [13]. Such findings support the development of responder phenotypes, in which patients are categorized according to clinically meaningful combinations of response domains rather than evaluated only through mean changes in isolated variables. This approach is particularly relevant for precision diabetology, because it may help identify which patients obtain concordant metabolic benefit, which patients exhibit discordant responses, and which patients may require treatment adjustment or additional lifestyle, nutritional, or functional interventions [14].

From a systems perspective, this shift is biologically coherent. Insulin is not an isolated regulator of blood glucose, but part of a highly integrated metabolic ecosystem. Its actions intersect with adipose tissue biology, skeletal muscle glucose uptake, hepatic glucose production, pancreatic beta-cell reserve, incretin signaling, renal glucose handling, inflammatory pathways, vascular function, and central mechanisms regulating appetite and energy balance [15]. When several components of this ecosystem are impaired simultaneously, as commonly occurs in long-standing obesity-associated T2DM, diagnosis and treatment cannot be adequately conceptualized through one marker alone [15,16]. HbA1c remains essential, but it should be interpreted together with adiposity, body composition, inflammatory status, renal stability, and functional preservation [16].

In this context, patient-level multidimensional response analysis offers a clinically interpretable framework for evaluating therapeutic benefit. Instead of asking only which treatment lowers HbA1c more, this approach asks what type of response each patient develops. A glycemic-only response may indicate effective glucose lowering without improvement in the adiposity-driven component of disease. An adiposity-only response may suggest metabolic remodeling that is not yet reflected in glycemic control. A concordant glycemic–adiposity response may indicate broader therapeutic benefit, while a multidimensional response that also includes inflammatory improvement and the preservation of muscle strength may represent a higher-quality response phenotype. Conversely, a low or absent response across domains may identify patients who require closer reassessment [17].

The present study was designed as a secondary patient-level analysis of a real-world cohort of adults with T2DM followed for 12 months in a tertiary diabetes center in Eastern Europe. Building on paired baseline and follow-up assessments, we classified patients according to individual response phenotypes across glycemic and adiposity domains, and further constructed a multidimensional response score incorporating glycemic response, fat mass reduction, hs-CRP reduction, and the preservation of handgrip strength. The aim was not simply to repeat treatment–class comparisons, but to characterize concordant and discordant patterns of therapeutic response in a metabolically complex population.

We hypothesized that the response to antidiabetic therapy would be heterogeneous and that clinically meaningful discordant phenotypes would be identifiable. Specifically, we expected that some patients would achieve glycemic improvement without adiposity response, whereas others would show broader multidimensional improvement. By focusing on responder phenotypes rather than average treatment effects alone, this analysis seeks to contribute to a more integrated interpretation of therapeutic response in T2DM and to support the development of more individualized strategies for obesity-associated diabetes care. To keep the aims focused, the primary objective of this analysis was defined a priori as characterizing the distribution of concordant and discordant glycemic–adiposity response phenotypes and the frequency of high multidimensional response, and secondarily as describing how these phenotypes were distributed across stable metformin-based treatment classes; all remaining analyses, including exploratory predictors, were considered hypothesis-generating.

## 2. Materials and Methods

### 2.1. Study Design and Setting

This study was designed as a secondary patient-level response-heterogeneity analysis of a prospective real-world observational cohort of adults with type 2 diabetes mellitus (T2DM). The primary purpose of the present analysis was not to re-estimate mean treatment effects across antidiabetic classes, but to classify individual patients according to clinically interpretable patterns of glycemic, adiposity, inflammatory, and functional response over 12 months.

The cohort was recruited and followed at the National Institute of Diabetes, Nutrition and Metabolic Diseases, a tertiary diabetes referral center in Bucharest, Romania. Baseline assessments were performed during routine outpatient care between June and December 2024, and follow-up assessments were completed after 12 months, between June and December 2025. Treatment allocation was not randomized and reflected routine clinical decision-making. Therefore, all treatment-related comparisons were interpreted as observational associations rather than causal effects.

Patients were managed according to standard institutional diabetes care, with routine outpatient review approximately every three months. Anthropometry, body composition analysis, handgrip dynamometry, and the full laboratory panel were obtained at the baseline and 12-month study visits, whereas interim visits focused on glycemic monitoring and treatment safety. As part of routine care, all patients had access to qualified lifestyle support, including structured dietary and physical activity counseling delivered by diabetologists and dietitians; the intensity of, and adherence to, this advice were not systematically quantified, as is noted among the study limitations.

### 2.2. Study Population and Analytic Cohort

Adults with confirmed T2DM were screened for eligibility. Patients were considered eligible if they were receiving metformin monotherapy or metformin-based therapy combined with one predefined second-line antidiabetic class and had paired baseline and 12-month follow-up data. Patients were required to remain on the same antidiabetic regimen during the observation period in order to allow the classification of responses under stable treatment exposure.

Exclusion criteria included complex or unclassifiable antidiabetic regimens, continuous triple therapy outside the predefined analytic framework, severe anemia (hemoglobin < 11 g/dL), severe hypertriglyceridemia (triglycerides > 400 mg/dL), duplicate records, missing baseline data, incomplete key variables, treatment change during follow-up, withdrawal, loss to follow-up, or absence of 12-month assessment.

Of 291 screened adults with T2DM, 202 met eligibility criteria. Among these, 166 patients completed 12-month follow-up without treatment change and had the variables required for patient-level response classification. These 166 patients constituted the final analytic cohort for the present responder-phenotype analysis.

The reduction from 291 screened to 166 analyzed patients reflects two sequential steps rather than treatment failure or tolerability-driven discontinuation. First, 89 patients were excluded at eligibility because they did not fit the predefined single-add-on framework (complex, unclassifiable, or continuous triple-therapy regimens), had severe anemia or severe hypertriglyceridemia, were duplicate records, or had missing baseline data. Second, of the 202 eligible patients, a further 36 were excluded because they did not complete the 12-month assessment on an unchanged regimen (treatment change or intensification during follow-up, withdrawal, loss to follow-up, or incomplete key variables). The dominant driver was therefore methodological: the responder-phenotype design required a stable, classifiable, metformin-based exposure for the full 12 months, and in routine care a substantial proportion of patients underwent treatment intensification or switching, which necessarily removed them from a stable exposure analysis. This is described further as a potential source of selection bias in the Limitations.

All participants provided written informed consent. The study was conducted in accordance with the Declaration of Helsinki and was approved by the Ethics Committee of the Carol Davila University of Medicine and Pharmacy, Bucharest (approval number 15142, dated 3 June 2022).

### 2.3. Treatment Exposure

The antidiabetic regimen maintained throughout the 12-month observation period was used as the treatment exposure variable. Patients were classified into six mutually exclusive treatment groups, as follows:

- metformin monotherapy (*N* = 46);
- metformin plus sulfonylurea (*N* = 10);
- metformin plus dipeptidyl peptidase-4 inhibitor (*N* = 17);
- metformin plus sodium-glucose cotransporter-2 inhibitor (*N* = 25);
- metformin plus glucagon-like peptide-1 receptor agonist (*N* = 23);
- metformin plus insulin (*N* = 45).

In the present analysis, treatment class was used primarily to describe the distribution of response phenotypes across therapeutic regimens. The main unit of analysis was the individual patient response pattern rather than the mean treatment contrast.

Within each add-on class, agents were prescribed and titrated at standard clinical doses according to routine practice and national prescribing conditions, and metformin was continued as background therapy in every non-monotherapy group; thus all treatment arms shared a metformin backbone, with metformin monotherapy serving as the common reference.

### 2.4. Clinical, Anthropometric, Functional, and Laboratory Assessments

Clinical and laboratory assessments were performed at baseline and at 12 months. Baseline variables included age, sex, diabetes duration, smoking status, hypertension, cardiovascular disease, dyslipidemia, statin use, ACE inhibitor or angiotensin receptor blocker use, recent infection, anti-inflammatory drug use, glucocorticoid use, physical activity level, resistance training, and available adherence indicators.

Anthropometric assessment included body weight, height, body mass index (BMI), waist circumference, hip circumference, and waist-to-height ratio. Body composition was assessed using a Fresenius Body Composition Monitor, with patients examined in the supine position according to standard device procedures [18,19]. Fat mass percentage, absolute fat mass, lean mass, and skeletal muscle mass were recorded when available.

Muscle strength was assessed using a Saehan hydraulic handgrip dynamometer. Measurements were performed on the dominant hand, and the best value from three maximal voluntary contractions was used for analysis [20].

Glycemic and biochemical assessments included HbA1c, fasting plasma glucose, insulin, C-peptide, HOMA-IR, lipid profile, serum creatinine, estimated glomerular filtration rate (eGFR), urea, urinary albumin, and albumin-to-creatinine ratio. HbA1c was measured by high-performance liquid chromatography. Other biochemical variables were measured using standardized automated methods in the institution’s certified laboratory. The lipid profile comprised total cholesterol, LDL cholesterol, HDL cholesterol, and triglycerides; statin use, antihypertensive therapy (including ACE inhibitors or angiotensin-receptor blockers), and a diagnosis of hypertension or dyslipidemia were also recorded at baseline, so that the broader cardiovascular risk profile, and not only glycemia, was characterized.

Inflammatory status was assessed using erythrocyte sedimentation rate, C-reactive protein, high-sensitivity C-reactive protein (hs-CRP), fibrinogen, and white blood cell count. hs-CRP was used as the principal marker of low-grade systemic inflammation in the responder-phenotype analysis [21].

### 2.5. Definition of Change Variables

For each patient, absolute change from baseline to 12 months was calculated as the 12-month value minus the baseline value. Therefore, negative values for HbA1c, body weight, fat mass, and hs-CRP indicated improvements. Relative percentage change was calculated as absolute change divided by the baseline value and multiplied by 100.

For HbA1c, response was defined using absolute percentage point reduction. For fat mass and hs-CRP, response was defined using relative percentage reduction. Handgrip strength was treated differently because the relevant clinical question was not improvement but preservation of function during metabolic and adiposity reduction.

### 2.6. Primary Glycemic–Adiposity Response Phenotypes

The primary responder classification crossed two clinically relevant domains, namely, glycemic response and adiposity response.

Glycemic response was defined as an absolute HbA1c reduction of at least 0.5 percentage points from baseline to 12 months.

Adiposity response was defined as a relative reduction in absolute fat mass of at least 5% from baseline to 12 months.

Using these two domains, patients were classified into four mutually exclusive response phenotypes:

- Glycemic–adiposity responders—patients achieving both HbA1c reduction ≥ 0.5 percentage points and fat mass reduction ≥ 5%;
- Glycemic-only responders—patients achieving HbA1c reduction ≥ 0.5 percentage points without fat mass reduction ≥ 5%;
- Adiposity-only responders—patients achieving fat mass reduction ≥ 5% without HbA1c reduction ≥ 0.5 percentage points;
- Low/non-responders—patients achieving neither HbA1c reduction ≥ 0.5 percentage points nor fat mass reduction ≥ 5%.

The glycemic-only and adiposity-only categories were considered discordant response phenotypes, because they identified patients in whom improvement occurred in one major therapeutic domain but not the other.

### 2.7. Multidimensional Response Domains and Score

To capture response quality beyond the glycemic–adiposity axis, a four-domain multidimensional response score was constructed. One point was assigned for each of the following domains:

- Glycemic response —HbA1c reduction ≥ 0.5 percentage points;
- Adiposity response—relative fat mass reduction ≥ 5%;
- Inflammatory response—relative hs-CRP reduction ≥ 20%;
- Functional preservation—handgrip strength decline no greater than 1 kg from baseline to 12 months.

The multidimensional response score therefore ranged from 0 to 4. A score of 0 indicated the absence of response in all four domains, whereas a score of 4 indicated simultaneous glycemic response, adiposity response, inflammatory response, and the preservation of muscle strength.

A high multidimensional response was defined as a score of at least 3. A complete multidimensional response was defined as a score of 4.

Renal stability was assessed separately and was not included in the core multidimensional score, because eGFR remained broadly stable in the cohort and showed limited discriminatory capacity as a responder domain. Renal stability was defined descriptively as the absence of clinically relevant eGFR decline during follow-up.

### 2.8. Study Outcomes

The primary outcome of the present analysis was the distribution of glycemic–adiposity response phenotypes in the overall cohort and across treatment classes.

Secondary outcomes included the following:

- Distribution of individual response domains by treatment class;
- Distribution of the multidimensional response score by treatment class;
- Proportion of patients achieving high multidimensional response;
- Proportion of patients achieving complete multidimensional response;
- Baseline clinical and metabolic characteristics according to response phenotype;
- Twelve-month changes in glycemic, anthropometric, body-composition, inflammatory, functional, and renal variables according to response phenotype;
- Exploratory predictors of high multidimensional response.

### 2.9. Statistical Analysis

Continuous variables were summarized as mean ± standard deviation or median with interquartile range, depending on distribution. Categorical variables were summarized as counts and percentages. Baseline characteristics were described for the full cohort and according to the four primary glycemic–adiposity response phenotypes.

Between-phenotype comparisons for continuous variables were performed using Kruskal–Wallis tests, given the modest sample size and unequal group distribution. Categorical variables were compared using chi-square tests or Fisher exact tests where appropriate. For sparse contingency tables, exact or permutation-based *p*-values were preferred, and the results were interpreted descriptively.

The primary analysis was descriptive and phenotype-based. The proportion of patients in each glycemic–adiposity response phenotype was calculated overall and within each treatment class. Stacked bar plots were used to visualize treatment-specific distributions of glycemic–adiposity phenotypes. Individual-level changes in HbA1c and fat mass were visualized using a quadrant plot, with the vertical and horizontal threshold lines corresponding to an HbA1c reduction ≥ 0.5 percentage points and a fat mass reduction ≥ 5%, respectively.

The four-domain multidimensional response score was summarized overall and by treatment class. Heatmaps were used to display the percentage of patients achieving each response domain within each treatment group. The distribution of response domain combinations was also described to identify concordant and discordant patterns across glycemic, adiposity, inflammatory, and functional domains.

Exploratory logistic regression analyses were used to evaluate potential predictors of high multidimensional response, defined as a multidimensional score ≥ 3. Candidate predictors included age, sex, diabetes duration, baseline HbA1c, baseline BMI, baseline fat mass percentage, baseline hs-CRP, baseline handgrip strength, treatment class, adherence indicators, and resistance training. Continuous predictors were standardized and reported per one standard deviation increase. Because of the limited sample size, sparse cells, and risk of separation, these models were interpreted as exploratory hypothesis-generating analyses rather than confirmatory predictive models.

No missing values were imputed. Analyses were performed using the available complete data for each specified endpoint. All tests were two-sided, and *p*-values < 0.05 were considered nominally significant. Given the exploratory nature of the secondary responder phenotype analysis, emphasis was placed on effect size, pattern consistency, and clinical interpretability rather than on formal multiplicity-driven claims.

## 3. Results

### 3.1. Study Population and Individual Response-Domain Overview

The final analytic cohort included 166 adults with type 2 diabetes mellitus who completed 12-month follow-up without treatment change and had the variables required for patient-level response classification. The response was analyzed at the individual patient level rather than only as mean treatment group change. Four core response domains were evaluated, namely, glycemic response, adiposity response, inflammatory response, and functional preservation. Renal stability was assessed as a safety descriptor and was not incorporated into the core multidimensional response score because all patients met the prespecified renal-stability criterion.

Overall, 137 of 166 patients (82.5%) achieved glycemic response, defined as an HbA1c reduction of at least 0.5 percentage points. Adiposity response, defined as a fat mass reduction of at least 5%, occurred in 58 of 166 patients (34.9%). Inflammatory response, defined as an hs-CRP reduction of at least 20%, occurred in 73 of 166 patients (44.0%). Functional preservation was observed in 143 of 166 patients (86.1%), and all patients met the prespecified renal-stability criterion. High multidimensional response, defined as a score of at least three of four domains, occurred in 78 patients (47.0%), while complete multidimensional response across all four domains occurred in 25 patients (15.1%) (Table 1). The cohort was metabolically complex at baseline; patients had long-standing, poorly controlled disease (mean baseline HbA1c 8.9%) with class II obesity (mean BMI 36.3 kg/m^2^), and a high burden of cardiovascular risk factors including hypertension, dyslipidemia, and statin use, with baseline characteristics broadly comparable across response phenotypes.

### 3.2. Primary Glycemic–Adiposity Response Phenotypes

The cross-classification of glycemic response and adiposity response identified four mutually exclusive patient-level phenotypes. Glycemic–adiposity response was present in 49 patients (29.5%). The largest group was composed of glycemic-only responders, accounting for 88 patients (53.0%). Adiposity-only response was uncommon, occurring in 9 patients (5.4%), while 20 patients (12.0%) met neither the glycemic nor the adiposity response criterion. Thus, discordant response patterns were frequent, with most patients improving in HbA1c without achieving a parallel fat mass response.

**Figure 1:**
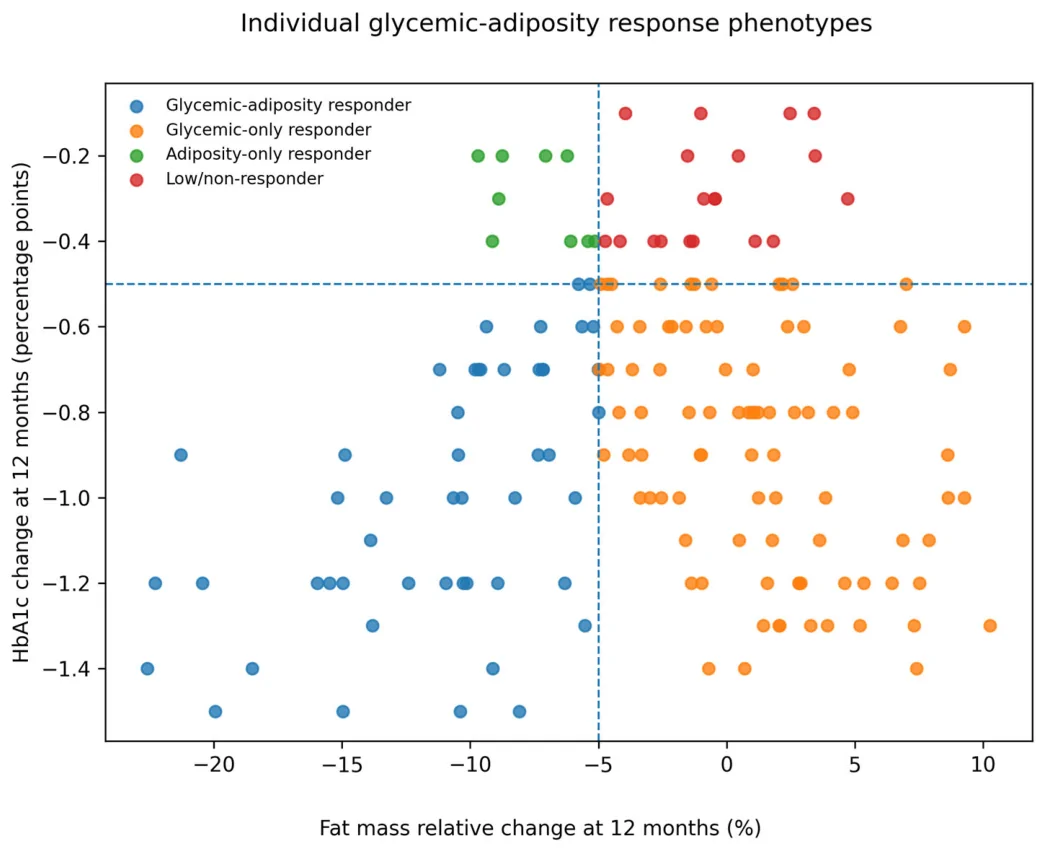
Individual glycemic–adiposity response quadrant plot. Each point represents one patient. The horizontal threshold indicates an HbA1c reduction of 0.5 percentage points, and the vertical threshold indicates a fat mass reduction of 5%. The lower left quadrant represents glycemic–adiposity response; the lower right quadrant represents glycemic-only response; the upper left quadrant represents adiposity-only response; and the upper right quadrant represents low/non-response.

**Figure 2:**
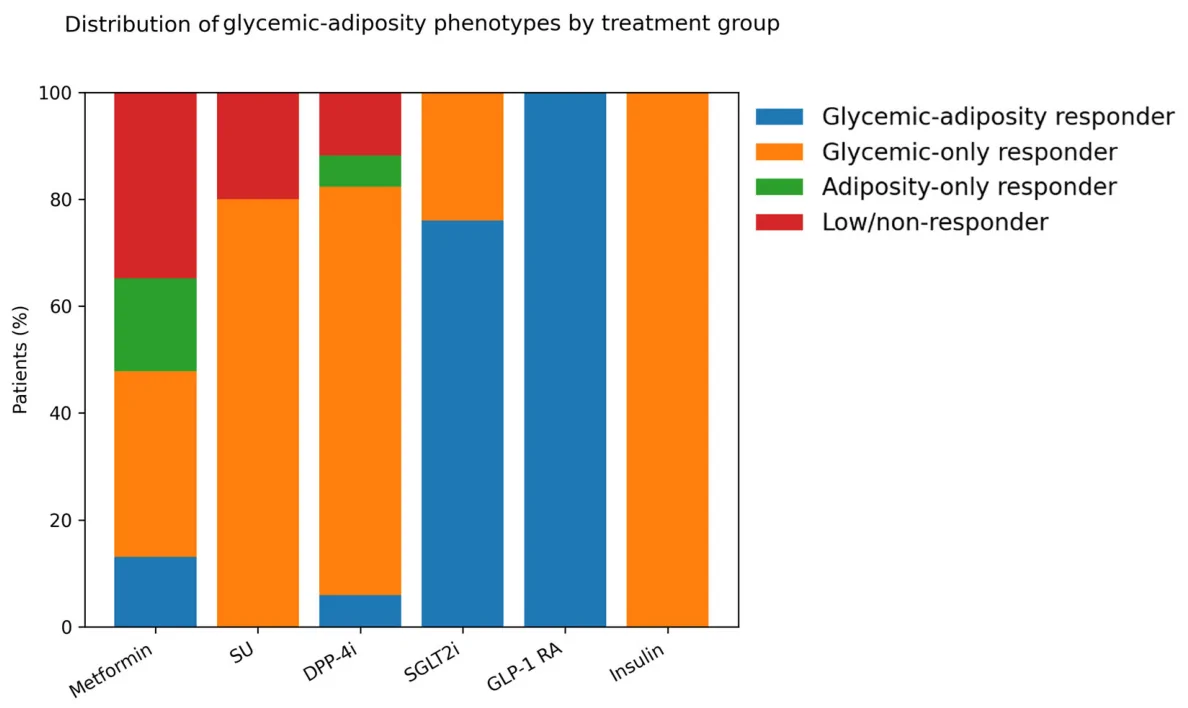
Distribution of glycemic–adiposity response phenotypes by treatment group. Stacked bars show the percentage distribution of the four primary response phenotypes within each treatment class, illustrating the enrichment of the concordant glycemic–adiposity response in GLP-1 RA and SGLT2i groups and the enrichment of glycemic-only response in the insulin group.

The distribution of response phenotypes differed markedly across treatment classes (global permutation *p* < 0.001). All patients treated with GLP-1 receptor agonists were classified as glycemic–adiposity responders (23/23, 100.0%). Most patients treated with SGLT2 inhibitors were also glycemic–adiposity responders (19/25, 76.0%), while the remaining SGLT2 inhibitor-treated patients were glycemic-only responders. By contrast, all insulin-treated patients were classified as glycemic-only responders (45/45, 100.0%), reflecting improvements in HbA1c without the prespecified fat mass response. Metformin monotherapy showed the broadest heterogeneity, including glycemic–adiposity response, glycemic-only response, adiposity-only response, and low/non-response (Table 2; Figure 1 and Figure 2).

### 3.3. Baseline Characteristics According to Response Phenotype

Baseline demographic, metabolic, body composition, functional, inflammatory, renal, and lifestyle characteristics were generally comparable across the four primary response phenotypes. No statistically significant between-phenotype differences were observed for age, diabetes duration, baseline HbA1c, body weight, BMI, fat mass percentage, absolute fat mass, handgrip strength, hs-CRP, eGFR, medication adherence, sex, hypertension, dyslipidemia, statin use, or resistance training. This suggests that the observed response categories were not explained by a single dominant baseline characteristic in this cohort (Table 3).

### 3.4. Twelve-Month Changes According to Response Phenotype

Longitudinal change profiles confirmed the clinical distinction between the four response phenotypes. Glycemic–adiposity responders showed simultaneous improvement in HbA1c and fat mass, with mean HbA1c changes of −1.00 percentage points and mean fat mass changes of −4.4 kg, corresponding to an average relative fat mass reduction of 11.0%. This group also showed the largest relative hs-CRP reduction (−30.8%).

Glycemic-only responders achieved substantial HbA1c improvement (mean change −0.89 percentage points) but did not achieve adiposity response; on average, body weight and fat mass increased slightly in this group. Adiposity-only responders showed fat mass reductions (mean change −2.9 kg; relative change −7.4%) without clinically meaningful HbA1c reductions (mean change −0.30 percentage points). Low/non-responders showed only modest changes in both HbA1c and fat mass. Between-phenotype differences were significant for HbA1c, body weight, fat mass indices, hs-CRP relative change, and multidimensional score, whereas handgrip and eGFR changes remained similar across phenotypes (Table 4).

### 3.5. Multidimensional Response Score and Response Domain Combinations

The multidimensional response score further separated treatment-associated response patterns. GLP-1 RA therapy showed the highest multidimensional response profile, with all patients achieving high multidimensional response and 52.2% achieving complete multidimensional response. SGLT2 inhibitor therapy also showed a favorable multidimensional pattern, with 80.0% high multidimensional response and 40.0% complete multidimensional response. In contrast, complete multidimensional response was absent in the SU and insulin groups and uncommon with metformin and DPP-4 inhibitor therapy (Table 5).

**Figure 3:**
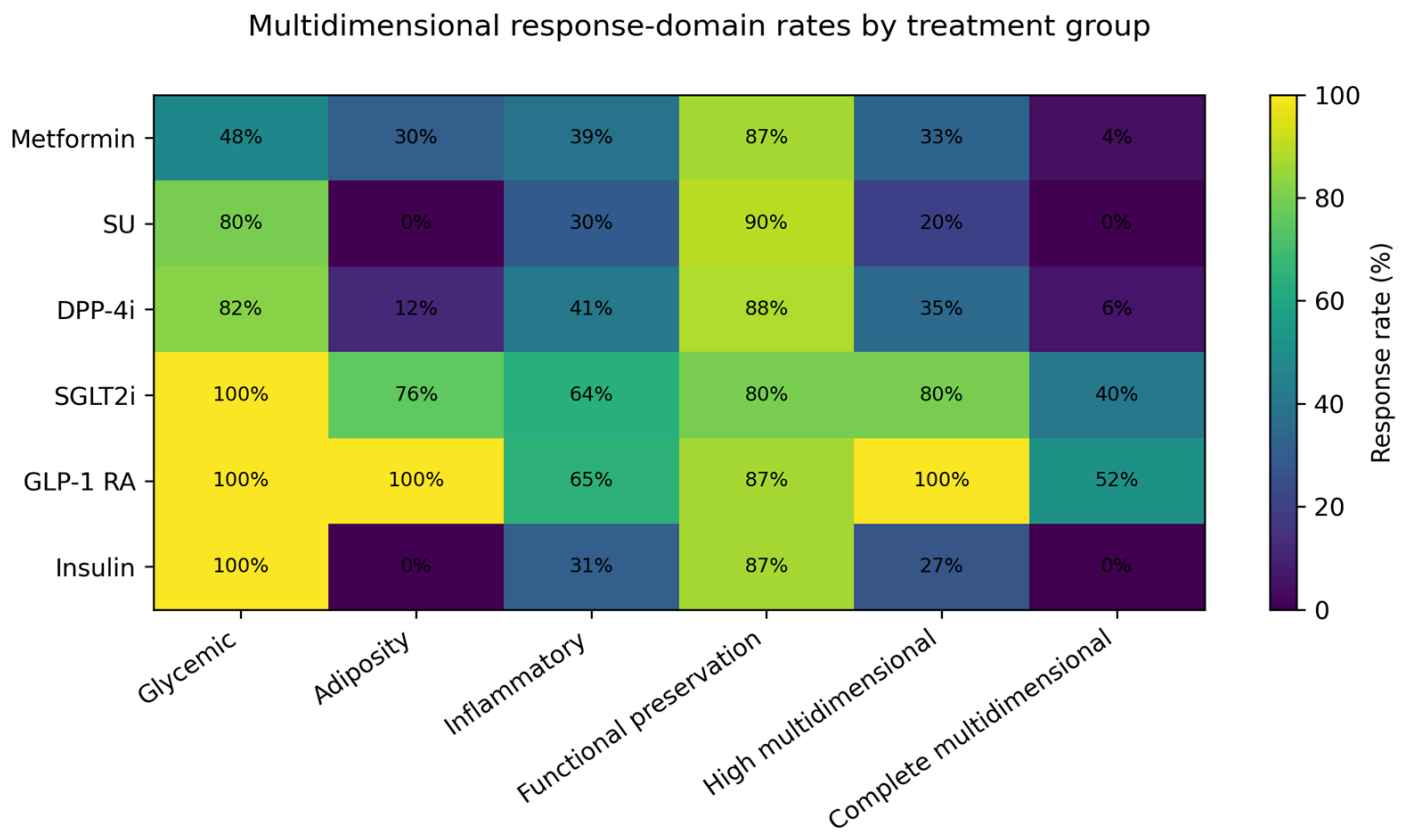
Multidimensional response domain rates by treatment group. The heatmap displays the percentage of patients in each treatment group within each individual response domain, as well as high and complete multidimensional response.

**Figure 4:**
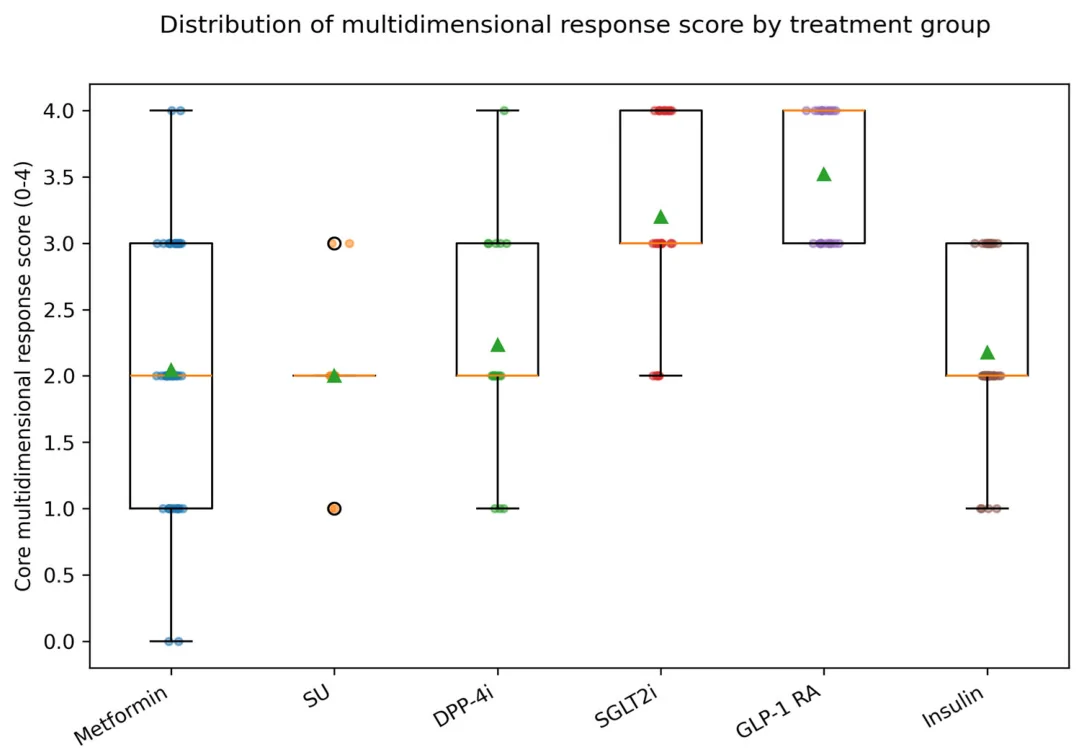
Multidimensional response score distribution by treatment group. Bars show the distribution of scores from 0 to 4 within each treatment group. The GLP-1 RA and SGLT2i groups showed the highest frequency of scores of 3 or 4.

The response domain heatmap shows that functional preservation was frequent across all treatment classes, whereas adiposity response was the most discriminating domain. Glycemic response was frequent in all add-on treatment groups, but adiposity response occurred almost exclusively among GLP-1 RA and SGLT2 inhibitor-treated patients. Inflammatory response was more common in the GLP-1 RA and SGLT2 inhibitor groups than in the remaining groups, although this domain remained exploratory (Figure 3 and Figure 4).

**Figure 5:**
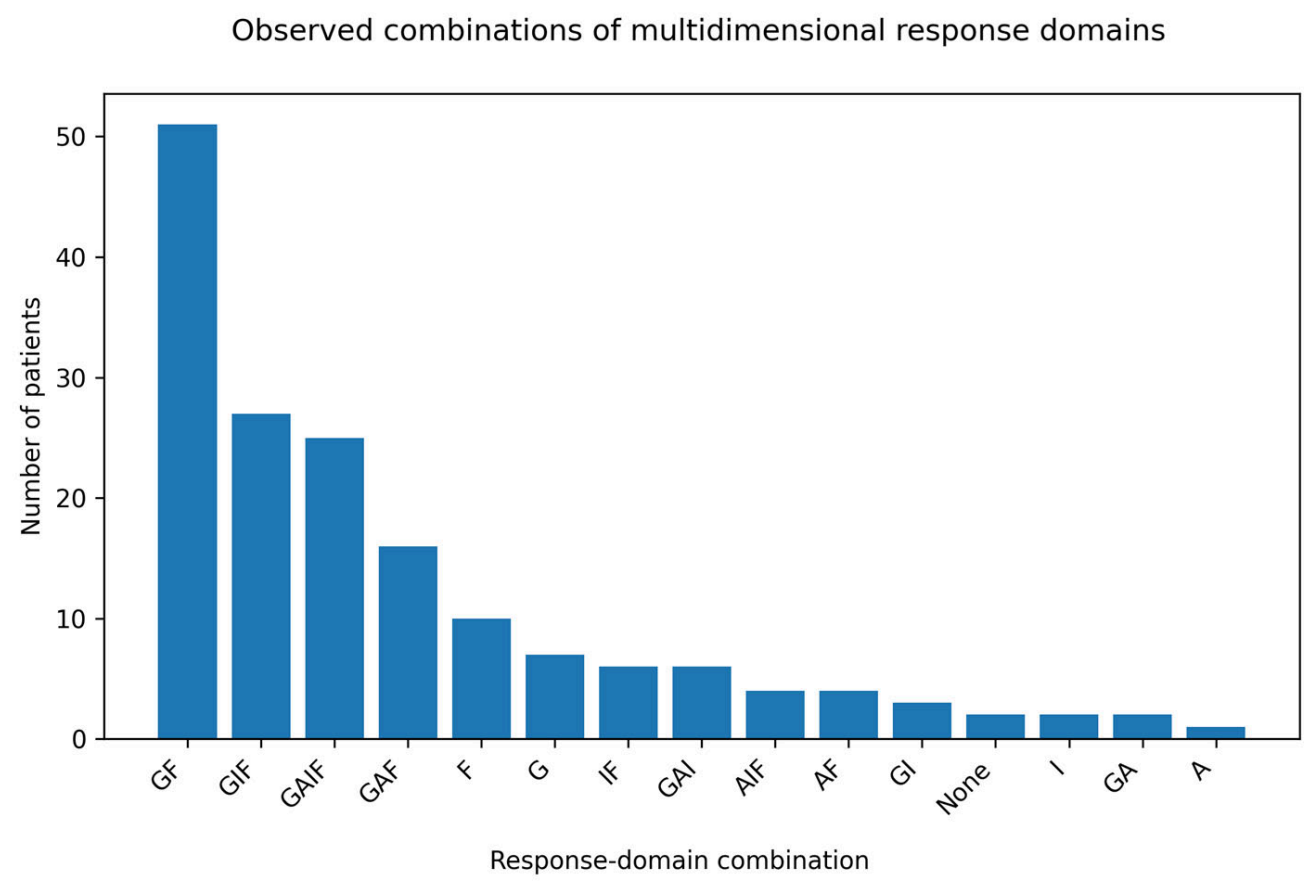
Bars show the frequency of each observed combination of glycemic, adiposity, inflammatory, and functional domains. The pattern emphasizes that response heterogeneity extended beyond the binary glycemic–adiposity classification.

When specific combinations of response domains were examined, the most common pattern was glycemic response with functional preservation, without adiposity or inflammatory response (51 patients, 30.7%). The second most common pattern was glycemic response, inflammatory response, and functional preservation (27 patients, 16.3%). Complete response across glycemic, adiposity, inflammatory, and functional domains occurred in 25 patients (15.1%), mostly in the GLP-1 RA and SGLT2 inhibitor groups. These patterns underscore that individual response quality was heterogeneous, and that glycemic improvement alone did not necessarily indicate a broader metabolic, inflammatory, and functional response (Table 6; Figure 5).

### 3.6. Exploratory Predictors of High Multidimensional Response

Exploratory univariable logistic models were used to identify baseline or treatment-associated factors related to high multidimensional response. Most baseline clinical variables, including age, diabetes duration, baseline HbA1c, BMI, fat mass percentage, handgrip strength, medication adherence, sex, and resistance training, were not significantly associated with high multidimensional response. Higher baseline ln(hs-CRP) was associated with lower odds of a high multidimensional response (OR 0.71 per 1 SD higher, 95% CI 0.52–0.98; *p* = 0.036).

**Figure 6:**
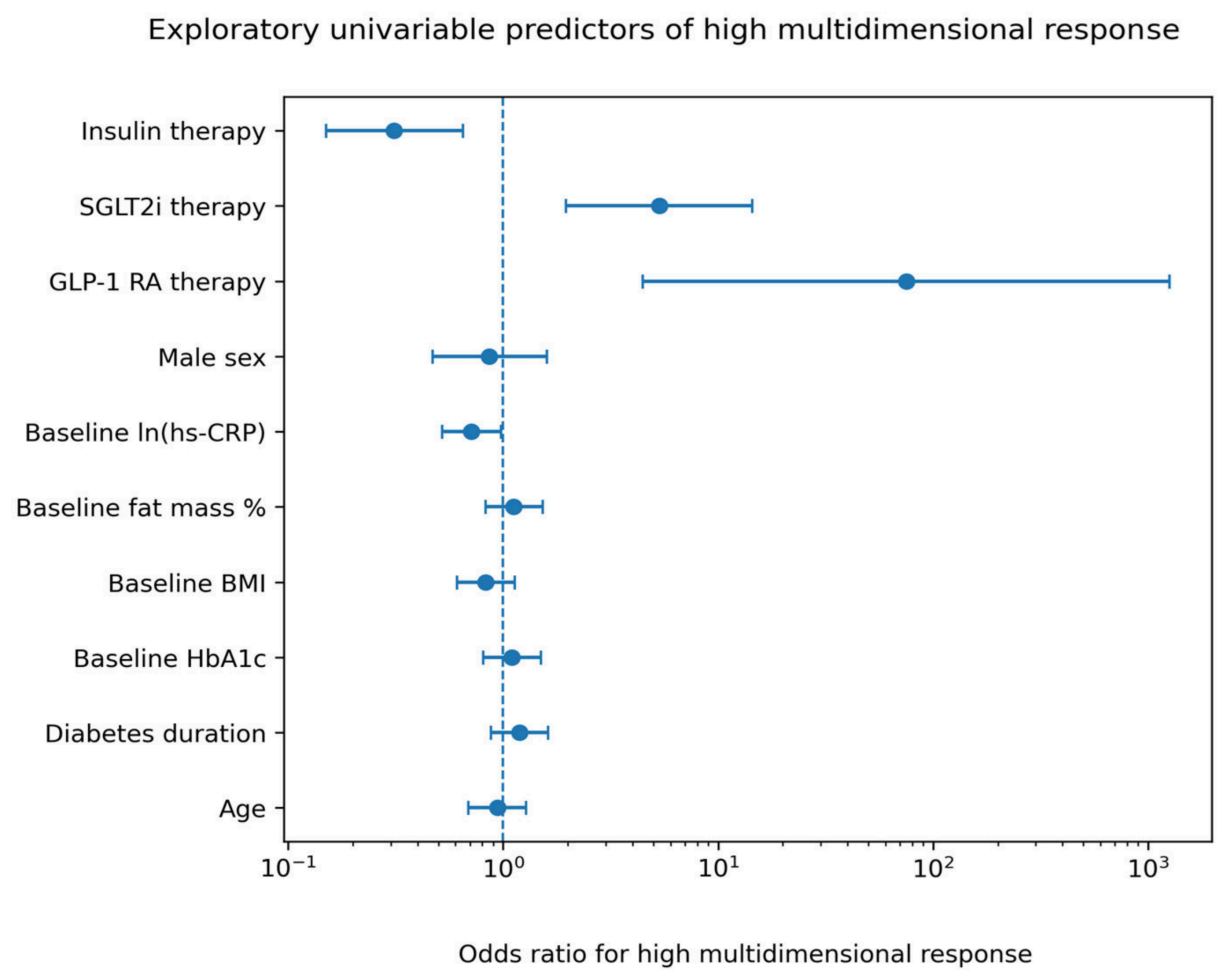
Exploratory univariable predictors of high multidimensional response. Points indicate odds ratios and horizontal lines indicate 95% confidence intervals on a logarithmic scale. The vertical reference line represents an odds ratio of 1.0.

Treatment class showed the strongest exploratory association with high multidimensional response. GLP-1 RA therapy was associated with markedly higher odds of high multidimensional response (OR 74.95, 95% CI 4.46–1258.90; *p* < 0.001), and SGLT2 inhibitor therapy was also positively associated with high multidimensional response (OR 5.32, 95% CI 1.96–14.44; *p* < 0.001). Insulin therapy was negatively associated with high multidimensional response (OR 0.31, 95% CI 0.15–0.65; *p* = 0.002). These models should be interpreted as exploratory because several treatment categories had sparse or complete response patterns, leading to wide confidence intervals and potential separation (Table 7; Figure 6).

### 3.7. Summary of the Response Phenotype Pattern

In summary, patient-level response heterogeneity was substantial. Although glycemic response was common, fewer than one third of patients achieved simultaneous glycemic and adiposity responses, and only 15.1% achieved complete multidimensional response across glycemic, adiposity, inflammatory, and functional domains. The most clinically relevant discordant pattern was glycemic-only response, particularly in the insulin group, whereas concordant glycemic–adiposity and high multidimensional responses were concentrated in the GLP-1 RA and SGLT2 inhibitor groups. These findings support the analysis of treatment response as a multidimensional patient-level phenotype rather than as isolated mean change in HbA1c or body weight.

## 4. Discussion

### 4.1. Principal Findings

This secondary patient-level analysis shows that therapeutic response in obesity-associated type 2 diabetes mellitus is substantially heterogeneous when response is evaluated across multiple clinically relevant domains rather than through mean HbA1c change alone, consistent with previous evidence on treatment response heterogeneity and precision diabetology in T2DM [11,12,14]. Although most patients achieved a glycemic response over 12 months, fewer than one third achieved a concordant glycemic–adiposity response, and only a minority achieved complete multidimensional response across glycemic, adiposity, inflammatory, and functional domains. The most frequent discordant phenotype was glycemic-only response, indicating that improvement in HbA1c often occurred without parallel improvement in fat mass. Conversely, an adiposity-only response was uncommon, suggesting that isolated fat mass reduction without a clinically meaningful glycemic response was less frequent in this cohort.

The distribution of response phenotypes differed clearly across antidiabetic treatment classes, but the emphasis of the present analysis is not the estimation of causal treatment effects. Rather, the findings identify clinically interpretable patient-level response patterns under stable real-world treatment exposure. GLP-1 receptor agonist therapy and SGLT2 inhibitor therapy were enriched for concordant glycemic–adiposity and high multidimensional response, whereas insulin therapy was characterized by a predominantly glycemic-only pattern, consistent with the divergent weight-related and cardiometabolic profiles of these therapeutic classes [9,10]. These observations support the central premise of the study, which is that in patients with type 2 diabetes and obesity, the quality of response cannot be adequately described by glycemic control alone [11,12,14]. The concentration of a concordant and high multidimensional response among GLP-1 receptor agonist and, to a lesser extent, SGLT2 inhibitor users is directionally consistent with current guideline emphasis on these classes in obesity-associated T2DM; however, because treatment was allocated by clinical indication rather than randomized, these data should be read as supporting, not by themselves establishing, a change in treatment algorithms, which requires confirmation in randomized trials and larger cohorts.

### 4.2. Response Heterogeneity and Discordant Phenotypes

The present findings are consistent with the broader concept that response to modern antidiabetic therapy is heterogeneous at the individual level [22,23]. Recent real-world registry evidence has shown that reductions in HbA1c and body weight after GLP-1 receptor agonist initiation do not necessarily occur in the same patient, with a substantial proportion of individuals showing improvement in only one domain or in neither domain [11]. Additional real-world data also indicate that many patients treated with once-weekly injectable GLP-1 receptor agonists do not simultaneously achieve conventional HbA1c and weight-loss targets [24]. Our analysis extends this framework in two ways. First, it replaces body weight alone with absolute fat mass response, thereby shifting the focus from crude weight reduction to body-composition response. Second, it adds inflammatory response and functional preservation as additional dimensions of response quality.

The predominance of glycemic-only response is clinically important. A patient may appear to respond well if HbA1c is considered the sole endpoint, yet still fail to improve adiposity, low-grade inflammation, or body-composition-related risk. This is particularly relevant in obesity-associated type 2 diabetes, where excess fat mass is not merely a background characteristic but a central component of the pathophysiological phenotype [25]. A glycemic-only response may therefore represent partial therapeutic success rather than comprehensive metabolic improvement. Such a pattern may be acceptable when the immediate therapeutic priority is glycemic stabilization, but it may be insufficient when the broader aim is cardiometabolic risk reduction, body-composition improvement, or long-term functional preservation [26].

The adiposity-only phenotype was uncommon, but conceptually relevant. It identifies patients in whom fat mass improved without a parallel HbA1c response. In clinical practice, such discordance may reflect lower baseline glycemic reversibility, advanced beta-cell dysfunction, adherence heterogeneity, differences in dietary or physical activity behavior, or treatment mechanisms that preferentially affect body composition [27,28]. Although this subgroup was small in the present cohort, its identification is useful because it illustrates that body composition benefit and glycemic benefit should be assessed as related but non-identical therapeutic dimensions [28].

### 4.3. Concordant Glycemic–Adiposity Response and Quality of Metabolic Improvement

Concordant glycemic–adiposity response was concentrated in patients treated with GLP-1 receptor agonists and, to a lesser extent, SGLT2 inhibitors. This pattern is biologically plausible. GLP-1 receptor agonists improve glycemia through glucose-dependent insulin secretion, the suppression of inappropriate glucagon secretion, delayed gastric emptying, and central appetite regulation, while also promoting clinically relevant reductions in body weight and adiposity [29,30,31]. SGLT2 inhibitors improve glycemia through urinary glucose excretion, and may reduce body weight and fat mass through negative energy balance, osmotic diuresis, and changes in substrate utilization [32,33,34]. In contrast, insulin therapy improves glycemia but is frequently associated with weight gain or limited adiposity reduction [35,36], which is consistent with the glycemic-only profile observed in this analysis. Improvement in HbA1c without a parallel adiposity response is thus an expected pattern for insulin and sulfonylurea therapy, in which glucose lowering is commonly achieved without, or even against, a favorable body composition change.

The novelty of the present analysis is not the confirmation that GLP-1 receptor agonists and SGLT2 inhibitors have favorable average metabolic profiles; this is already well established [37]. The added value is the demonstration that these therapies were associated with a higher probability of integrated patient-level response. In other words, the relevant question becomes not only which treatment lowers mean HbA1c or mean weight, but which patients achieve a coordinated response across glycemic, adiposity, inflammatory, and functional domains. This distinction is important for precision diabetology because mean treatment effects may conceal clinically meaningful inter-individual heterogeneity [38].

Using fat mass reduction rather than body weight reduction strengthens the clinical interpretation of the responder phenotype. Weight loss may reflect changes in fat mass, lean mass, water balance, or gastrointestinal content. By contrast, fat mass response more directly captures adiposity reduction, and is therefore more aligned with the pathophysiological burden of obesity-associated type 2 diabetes [39,40]. In this context, a glycemic–adiposity responder phenotype may be viewed as a marker of higher-quality metabolic response, especially when accompanied by inflammatory improvement and preserved muscle strength.

### 4.4. Inflammatory Response and Functional Preservation

The inclusion of hs-CRP response adds an inflammatory dimension to the assessment of therapeutic response. Low-grade inflammation is closely linked to adiposity, insulin resistance, endothelial dysfunction, and cardiometabolic risk in type 2 diabetes [41,42]. In the present cohort, inflammatory response was more frequent among patients treated with GLP-1 receptor agonists and SGLT2 inhibitors than among the remaining groups, and glycemic–adiposity responders showed the largest mean relative reduction in hs-CRP. These findings suggest that improvements in adiposity and improvements in inflammatory status may cluster in a subset of patients, although the observational design does not allow the separation of direct drug effects from adiposity-mediated effects.

At the same time, hs-CRP is a nonspecific biomarker. It can be influenced by adiposity, intercurrent infection, medication use, hepatic function, smoking, and other inflammatory conditions [43]. Therefore, hs-CRP response should be interpreted as an exploratory marker of low-grade systemic inflammation rather than as proof of a specific anti-inflammatory mechanism. Nevertheless, its inclusion is clinically useful because it broadens the concept of response beyond glucose and fat mass, and because residual inflammatory risk may remain relevant even when glycemic control improves [44].

Because the cohort combined type 2 diabetes, obesity, and a high prevalence of hypertension and dyslipidemia, these patients carry a substantially increased risk of atherosclerotic cardiovascular disease, for which chronic low-grade inflammation and adiposity are important drivers. This context is relevant to the treatment class patterns observed here, because GLP-1 receptor agonists and SGLT2 inhibitors exert pleiotropic vascular effects that extend beyond glycemic control. GLP-1 receptor agonists have been shown to protect the vascular endothelium and to attenuate multiple steps of atherogenesis, including oxidative stress, systemic inflammation, monocyte recruitment, and plaque development [45]. SGLT2 inhibitors similarly display anti-atherosclerotic and vasculoprotective properties, with favorable effects on arterial function, inflammation, oxidative stress, and plaque stability in addition to their metabolic actions [46]. The enrichment of concordant glycemic–adiposity and inflammatory response in these groups is therefore biologically coherent with their recognized cardioprotective profiles, although the present observational design cannot separate direct vascular drug effects from adiposity- and inflammation-mediated effects.

Functional preservation was frequent across treatment classes and did not appear to deteriorate in patients with glycemic–adiposity response. This is an important observation because contemporary weight-centered diabetes care increasingly raises the question of whether weight or fat mass reduction is accompanied by a loss of muscle function [47,48]. In this cohort, fat mass response was not associated with a parallel decline in handgrip strength, supporting the interpretation that the favorable adiposity pattern, particularly in the GLP-1 receptor agonist and SGLT2 inhibitor groups, did not translate to a detectable functional compromise over 12 months. However, handgrip strength captures only one component of physical function; future studies should include more comprehensive functional testing.

### 4.5. Baseline Predictors and Individualized Therapeutic Assessment

Most baseline demographic, metabolic, inflammatory, functional, and lifestyle variables did not clearly distinguish response phenotypes. This suggests that simple baseline descriptors such as age, sex, baseline HbA1c, BMI, or fat mass percentage were insufficient to classify patients reliably into response categories in this cohort, consistent with the broader concern that simple stratification approaches may not fully capture the heterogeneity of type 2 diabetes [49]. Treatment class showed the strongest exploratory association with high multidimensional response, whereas insulin therapy was negatively associated with an integrated response. These associations should not be interpreted causally because treatment was not randomized, treatment selection reflected clinical indication, and some response patterns produced sparse cells and wide confidence intervals.

The weak discrimination by baseline variables has practical implications. It suggests that the prediction of multidimensional response may require richer phenotyping than is available in routine clinical datasets, although the model-based use of routinely available clinical features may still contribute to individualized treatment selection [50,51]. Potentially relevant predictors may include detailed drug dose exposure, adherence trajectories, dietary intake, physical activity changes, resistance training volume, protein intake, baseline insulin secretory reserve, ectopic fat distribution, inflammatory cytokine profiles, incretin biology, renal glucose handling, and genetic or metabolomic markers [52,53]. The present analysis therefore supports a two-step precision care framework, as follows: first, define clinically meaningful response phenotypes; second, identify predictors of phenotype membership in larger and more deeply characterized cohorts [49,53].

### 4.6. Strengths of the Analysis

This study has several strengths. First, it evaluates response at the individual patient level rather than relying only on mean between-group differences. Second, it uses a multidimensional framework that integrates glycemic response, adiposity response, inflammatory response, and functional preservation. Third, it focuses on fat mass rather than body weight alone, thereby addressing the quality of weight-related response. Fourth, it includes real-world patients from an Eastern European tertiary diabetes center, a population that remains underrepresented in many randomized trials and large registries. Finally, the analysis explicitly identifies discordant phenotypes, which are often hidden in conventional analyses centered on average HbA1c or body weight change [11,12,49].

### 4.7. Limitations

Several limitations should be acknowledged. Thus, in this secondary exploratory analysis of an observational cohort, treatment allocation was not randomized. Therefore, the results describe associations between treatment exposure and response patterns, not causal treatment effects. Confounding by indication is likely, particularly because patients selected for insulin, GLP-1 receptor agonists, SGLT2 inhibitors, or other add-on therapies may differ in disease severity, obesity phenotype, previous treatment history, comorbidity profile, and clinician-perceived therapeutic priorities. In addition, the sample size was modest, and treatment groups were unbalanced. Some categories, especially the sulfonylurea and DPP-4 inhibitor groups, included few patients, and several response phenotypes contained sparse cells. This limits statistical power, increases the risk of unstable estimates, and explains the wide confidence intervals observed in exploratory logistic models. The finding that all GLP-1 receptor agonist-treated patients met the glycemic–adiposity response definition should therefore be interpreted cautiously, and requires external validation in larger cohorts. The relatively small GLP-1 receptor agonist and SGLT2 inhibitor groups also reflect the reimbursement environment in Romania during the study period, when national prescribing and reimbursement criteria for these classes (including specialist prescription and glycemic or body mass index thresholds) restricted their use; this reduced the number of eligible patients maintained on a stable regimen of these agents, and further limited statistical power for the corresponding subgroups.

The analytic cohort included only patients who completed 12-month follow-up without treatment change. This design improves exposure stability but may introduce selection bias by excluding patients who discontinued treatment, switched therapy, were lost to follow-up, or had incomplete data. Consequently, the results may overrepresent patients with better tolerability, adherence, follow-up availability, or clinical stability. That said, the responder thresholds were clinically interpretable but partly arbitrary. An HbA1c reduction of at least 0.5 percentage points and a fat mass reduction of at least 5% are reasonable thresholds, but alternative cutoffs could produce different phenotype distributions. Similarly, an hs-CRP reduction of at least 20% and handgrip decline no greater than 1 kg should be viewed as exploratory operational definitions rather than validated outcome standards. Sensitivity analyses using alternative thresholds are therefore important for assessing robustness. In addition, although lipid parameters, statin use, and antihypertensive therapy were recorded at baseline, longitudinal changes in the lipid profile and in blood-pressure-lowering medication were not a prespecified component of this responder analysis, and warrant dedicated evaluation; likewise, response was assessed only at 12 months, so earlier changes at 3 or 6 months, and any subsequent attenuation of an initial response, could not be characterized.

Body composition was assessed using bioimpedance rather than dual-energy X-ray absorptiometry, computed tomography, or magnetic resonance imaging. Although bioimpedance is practical in routine care, it is affected by hydration status, edema, renal function, recent food intake, and device-specific algorithms [18,19]. Moreover, fat mass, lean mass, and skeletal muscle mass estimates do not capture ectopic fat, visceral adiposity, or muscle quality. Also, lifestyle and exposure variables were incompletely characterized. Nutritional intake, caloric restriction, protein intake, resistance training intensity, spontaneous physical activity, treatment dose escalation, drug persistence, and detailed adherence trajectories were not systematically quantified for all patients. These factors may substantially influence glycemic, adiposity, inflammatory, and functional outcomes [47,48,52].

Finally, hs-CRP was used as the principal inflammatory marker. Although clinically accessible, hs-CRP is nonspecific and cannot identify the cellular or molecular source of inflammation [43,44]. The absence of IL-6, TNF-alpha, adiponectin, leptin, and other inflammatory or adipokine markers limits mechanistic interpretation. In addition, the follow-up duration was limited to 12 months, and the study did not evaluate hard cardiovascular, renal, microvascular, or functional outcomes.

### 4.8. Perspectives

Future studies should validate this responder phenotype framework in larger prospective multicenter cohorts with prespecified definitions and the adequate representation of each treatment class [49,50]. Replication is particularly important for the GLP-1 receptor agonist and SGLT2 inhibitor patterns observed here, as well as for the low/non-responder and adiposity-only subgroups. External validation should also examine whether similar phenotypes are observed with newer incretin-based therapies, including dual GIP/GLP-1 receptor agonists, and whether phenotype membership predicts long-term cardiometabolic outcomes.

A major next step is the development of predictive models for multidimensional response. Such models should integrate routine clinical variables with deeper phenotyping, including drug dose and persistence, diet and physical activity data, body composition imaging, insulin secretion and insulin resistance markers, inflammatory panels, metabolomics, renal parameters, and possibly genetic or microbiome data [50,51,52,53]. Early on-treatment changes, for example at 3 or 6 months, may also help identify patients who are likely to become glycemic-only responders, concordant glycemic–adiposity responders, or low/non-responders at 12 months.

The clinical relevance of discordant phenotypes should also be tested prospectively. Patients with glycemic-only response may require additional weight-centered or adiposity-centered interventions, such as intensification to incretin-based therapy, structured nutrition programs, resistance training, or combined pharmacological strategies. Patients with adiposity-only response may require the reassessment of glycemic treatment intensity, insulin secretory capacity, or adherence. Low/non-responders may benefit from early therapeutic reconsideration rather than delayed escalation after prolonged exposure to ineffective regimens.

Last but not least, the concept of response quality should be incorporated into future diabetes outcomes research. A treatment response that improves HbA1c but worsens adiposity or fails to reduce inflammatory burden is not equivalent to a response that improves glycemia, reduces fat mass, lowers hs-CRP, and preserves muscle strength. Multidimensional responder phenotypes may therefore provide a clinically intuitive bridge between conventional therapeutic monitoring and precision diabetology [14,22,23,49].

## 5. Conclusions

In obesity-associated type 2 diabetes mellitus, therapeutic response is multidimensional and frequently discordant. Although glycemic improvement was common, concordant glycemic–adiposity response and complete multidimensional response were achieved by only a minority of patients, so response quality cannot be adequately judged from HbA1c change alone.

Regarding real-world practice, all this argues for appraising treatment success simultaneously across glycemic, adiposity, inflammatory, and functional domains. Patient-level responder phenotyping could help identify glycemic-only responders, most often on insulin, who may benefit from adiposity-directed intensification, while ensuring that muscle function is preserved during weight loss. The most integrated responses clustered with GLP-1 receptor agonist and, less uniformly, SGLT2 inhibitor therapy, consistent with their broader cardiometabolic profiles, although the observational design precludes causal inference.

Prospective, multicenter studies with prespecified response definitions are needed to validate these phenotypes and to test whether early responder classification predicts long-term cardiovascular, renal, inflammatory, and functional outcomes, and can thereby support more individualized treatment sequencing in obesity-associated diabetes.

## Figures and Tables

**Table 1:** Response domain rates and multidimensional response by treatment group.

Treatment Group	*N*	Glycemic *n* (%)	Adiposity *n* (%)	hs-CRP *n* (%)	Handgrip Preserved *n* (%)	High MRS *n* (%)	Complete MRS *n* (%)	MRS Mean ± SD
Metformin	46	22/46 (47.8%)	14/46 (30.4%)	18/46 (39.1%)	40/46 (87.0%)	15/46 (32.6%)	2/46 (4.3%)	2.0 ± 0.9
SU	10	8/10 (80.0%)	0/10 (0.0%)	3/10 (30.0%)	9/10 (90.0%)	2/10 (20.0%)	0/10 (0.0%)	2.0 ± 0.7
DPP-4i	17	14/17 (82.4%)	2/17 (11.8%)	7/17 (41.2%)	15/17 (88.2%)	6/17 (35.3%)	1/17 (5.9%)	2.2 ± 0.8
SGLT2i	25	25/25 (100.0%)	19/25 (76.0%)	16/25 (64.0%)	20/25 (80.0%)	20/25 (80.0%)	10/25 (40.0%)	3.2 ± 0.8
GLP-1 RA	23	23/23 (100.0%)	23/23 (100.0%)	15/23 (65.2%)	20/23 (87.0%)	23/23 (100.0%)	12/23 (52.2%)	3.5 ± 0.5
Insulin	45	45/45 (100.0%)	0/45 (0.0%)	14/45 (31.1%)	39/45 (86.7%)	12/45 (26.7%)	0/45 (0.0%)	2.2 ± 0.6
Global *p* (permutation/Kruskal)		<0.001	<0.001	0.026	0.965	<0.001	<0.001	

Values are *n*/*N* (%) unless otherwise stated. *N* = 166. Glycemic response = HbA1c reduction ≥ 0.5 percentage points; adiposity response = fat mass reduction ≥ 5%; inflammatory response = hs-CRP reduction ≥ 20%; functional preservation = handgrip decline no greater than 1 kg; high multidimensional response = MRS ≥ 3; complete multidimensional response = MRS = 4. Renal stability was 100% across all treatment groups and is described in the text. MRS, multidimensional response score; SU, sulfonylurea; DPP-4i, dipeptidyl peptidase-4 inhibitor; SGLT2i, sodium-glucose cotransporter-2 inhibitor; GLP-1 RA, glucagon-like peptide-1 receptor agonist.

**Table 2:** Primary glycemic–adiposity response phenotypes by treatment group.

Treatment Group	*N*	Glycemic–Adiposity Responder	Glycemic-Only Responder	Adiposity-Only Responder	Low/Non- Responder	Global Permutation *p*
Metformin	46	6/46 (13.0%)	16/46 (34.8%)	8/46 (17.4%)	16/46 (34.8%)	<0.001
SU	10	0/10 (0.0%)	8/10 (80.0%)	0/10 (0.0%)	2/10 (20.0%)	
DPP-4i	17	1/17 (5.9%)	13/17 (76.5%)	1/17 (5.9%)	2/17 (11.8%)	
SGLT2i	25	19/25 (76.0%)	6/25 (24.0%)	0/25 (0.0%)	0/25 (0.0%)	
GLP-1 RA	23	23/23 (100.0%)	0/23 (0.0%)	0/23 (0.0%)	0/23 (0.0%)	
Insulin	45	0/45 (0.0%)	45/45 (100.0%)	0/45 (0.0%)	0/45 (0.0%)	

Values are *n*/*N* (%). Glycemic–adiposity responders achieved both an HbA1c reduction ≥ 0.5 percentage points and a fat mass reduction ≥ 5%. Glycemic-only and adiposity-only responders represent discordant response phenotypes. The global *p*-value was obtained using permutation testing for the treatment-by-phenotype distribution.

**Table 3:** Baseline characteristics by primary glycemic–adiposity response phenotype.

Characteristic	Glycemic–Adiposity Responder (*N* = 49)	Glycemic-Only Responder (*N* = 88)	Adiposity-Only Responder (*N* = 9)	Low/Non- Responder (*N* = 20)	Global *p*
Age, years	58.5 ± 13.5; 58.0 [47.0; 68.0]	60.4 ± 13.3; 60.5 [50.0; 73.0]	56.1 ± 10.4; 57.0 [50.0; 61.0]	60.5 ± 11.2; 60.0 [53.0; 68.5]	0.703
Diabetes duration, years	14.8 ± 7.9; 15.8 [8.5; 20.5]	12.8 ± 7.4; 12.1 [7.1; 17.4]	16.4 ± 7.0; 17.0 [11.7; 18.6]	13.4 ± 8.4; 15.4 [5.8; 19.5]	0.321
HbA1c, %	8.9 ± 0.9; 8.8 [8.1; 9.8]	9.0 ± 1.0; 9.1 [8.3; 9.7]	8.8 ± 0.9; 8.6 [8.2; 9.2]	8.6 ± 0.9; 8.6 [8.1; 9.2]	0.387
Body weight, kg	102.6 ± 18.6; 103.0 [87.0; 115.0]	102.5 ± 16.9; 101.5 [91.6; 113.4]	98.0 ± 18.0; 104.2 [90.2; 109.4]	105.4 ± 20.1; 99.8 [94.3; 122.0]	0.886
BMI, kg/m^2^	35.5 ± 4.6; 35.1 [31.8; 38.7]	36.5 ± 4.2; 36.5 [33.3; 39.5]	35.6 ± 5.5; 34.4 [31.0; 40.8]	37.2 ± 4.1; 38.0 [34.2; 41.0]	0.387
Fat mass, %	40.1 ± 6.8; 38.2 [35.1; 44.7]	40.6 ± 6.1; 40.0 [37.5; 44.4]	40.5 ± 5.8; 39.4 [37.6; 41.7]	40.0 ± 5.0; 39.9 [36.4; 42.8]	0.894
Fat mass, kg	41.2 ± 10.3; 38.3 [33.6; 47.1]	41.5 ± 9.0; 40.9 [35.3; 48.4]	39.3 ± 7.2; 37.6 [37.0; 43.5]	41.9 ± 8.2; 42.0 [37.6; 46.8]	0.886
Handgrip strength, kg	31.0 ± 11.3; 29.8 [22.5; 39.9]	30.7 ± 9.4; 29.4 [23.6; 34.9]	30.1 ± 10.5; 29.5 [19.3; 38.2]	31.0 ± 10.3; 29.1 [23.3; 36.5]	0.998
hs-CRP, mg/L	4.9 ± 3.0; 4.5 [2.8; 6.0]	5.8 ± 2.9; 5.7 [3.5; 7.3]	5.7 ± 3.4; 5.9 [4.9; 6.2]	5.0 ± 3.2; 4.1 [3.0; 7.3]	0.144
eGFR, mL/min/1.73 m^2^	101.5 ± 11.3; 102.0 [93.0; 109.0]	99.6 ± 11.1; 100.0 [91.0; 109.0]	106.9 ± 11.8; 104.0 [102.0; 109.0]	100.1 ± 10.7; 100.5 [92.5; 106.5]	0.367
Medication adherence, %	89.0 ± 5.5; 89.0 [85.0; 93.0]	88.2 ± 6.3; 88.0 [83.0; 93.2]	91.0 ± 4.6; 90.0 [88.0; 95.0]	88.1 ± 5.8; 86.5 [83.0; 93.2]	0.509
Female sex	28/49 (57.1%)	53/88 (60.2%)	6/9 (66.7%)	12/20 (60.0%)	0.959
Hypertension	33/49 (67.3%)	58/88 (65.9%)	7/9 (77.8%)	13/20 (65.0%)	0.939
Dyslipidemia	36/49 (73.5%)	56/88 (63.6%)	5/9 (55.6%)	13/20 (65.0%)	0.618
Statin use	27/49 (55.1%)	61/88 (69.3%)	5/9 (55.6%)	11/20 (55.0%)	0.318
Resistance training	12/49 (24.5%)	20/88 (22.7%)	1/9 (11.1%)	6/20 (30.0%)	0.718

Continuous variables are shown as mean ± SD; median [IQR]. Categorical variables are shown as *n*/*N* (%). Between-phenotype comparisons used Kruskal–Wallis tests for continuous variables and chi-square or exact tests for categorical variables where appropriate. The table is restricted to core baseline variables relevant to response phenotype interpretation.

**Table 4:** Twelve-month changes and multidimensional response score by primary glycemic–adiposity response phenotype.

12-Month Change/ Score	Glycemic–Adiposity Responder	Glycemic-Only Responder	Adiposity-Only Responder	Low/Non- Responder	Global *p*
ΔHbA1c, percentage points	−1.00 ± 0.30; −1.00 [−1.20; −0.70]	−0.89 ± 0.27; −0.85 [−1.10; −0.67]	−0.30 ± 0.10; −0.30 [−0.40; −0.20]	−0.28 ± 0.12; −0.30 [−0.40; −0.20]	<0.001
Δbody weight, kg	−4.9 ± 1.9; −4.8 [−6.3; −3.2]	0.8 ± 1.9; 0.8 [−0.3; 2.2]	−2.5 ± 0.7; −2.7 [−3.1; −2.1]	−0.2 ± 1.1; 0.0 [−0.7; 0.6]	<0.001
Body-weight relative change, %	−4.9 ± 2.0; −4.6 [−6.1; −3.6]	0.9 ± 1.9; 0.8 [−0.3; 2.1]	−2.6 ± 0.6; −2.7 [−2.9; −2.3]	−0.2 ± 1.2; 0.0 [−0.8; 0.5]	<0.001
Δfat mass, kg	−4.4 ± 1.9; −3.7 [−5.9; −3.0]	0.5 ± 1.7; 0.5 [−0.7; 1.4]	−2.9 ± 1.0; −3.1 [−3.7; −2.0]	−0.3 ± 1.1; −0.3 [−1.0; 0.5]	<0.001
Fat mass relative change, %	−11.0 ± 4.8; −10.2 [−13.9; −7.3]	1.3 ± 4.0; 1.1 [−1.6; 3.7]	−7.4 ± 1.8; −7.1 [−8.9; −6.1]	−0.6 ± 2.8; −1.0 [−2.6; 1.3]	<0.001
Δfat mass, percentage points	−2.5 ± 1.2; −2.3 [−3.4; −1.6]	0.2 ± 1.0; 0.0 [−0.6; 0.8]	−1.9 ± 0.6; −2.0 [−2.3; −1.7]	−0.2 ± 0.8; −0.4 [−0.7; 0.2]	<0.001
hs-CRP relative change, %	−30.8 ± 28.6; −28.1 [−43.8; −8.9]	−9.7 ± 24.0; −11.2 [−26.3; 8.5]	−23.3 ± 25.4; −18.4 [−27.1; −11.1]	−11.3 ± 27.4; −12.4 [−24.1; 7.3]	<0.001
Δhandgrip, kg	0.7 ± 1.7; 0.8 [−0.3; 1.9]	0.7 ± 1.6; 0.6 [−0.2; 1.8]	0.7 ± 1.2; 1.0 [−0.1; 1.4]	0.4 ± 1.5; 0.8 [0.0; 1.3]	0.967
ΔeGFR, mL/min/1.73 m^2^	−0.3 ± 2.9; 0.0 [−3.0; 2.0]	0.1 ± 3.1; 0.0 [−3.0; 3.0]	1.0 ± 3.5; 2.0 [−1.0; 4.0]	−0.4 ± 3.1; −1.5 [−3.0; 2.2]	0.597
Multidimensional score, 0–4	3.5 ± 0.6; 4.0 [3.0; 4.0]	2.2 ± 0.6; 2.0 [2.0; 3.0]	2.3 ± 0.7; 2.0 [2.0; 3.0]	1.2 ± 0.6; 1.0 [1.0; 2.0]	<0.001

Values are mean ± SD; median [IQR]. Negative values for HbA1c, body weight, fat mass, and hs-CRP indicate improvement. The multidimensional score ranges from 0 to 4 and includes glycemic response, adiposity response, inflammatory response, and functional preservation.

**Table 5:** Distribution of multidimensional response score by treatment group.

Treatment Group	*N*	Score 0 *n* (%)	Score 1 *n* (%)	Score 2 *n* (%)	Score 3 *n* (%)	Score 4 *n* (%)	Score Mean ± SD	Score Median [IQR]	Global Kruskal *p*
Metformin	46	2/46 (4.3%)	11/46 (23.9%)	18/46 (39.1%)	13/46 (28.3%)	2/46 (4.3%)	2.0 ± 0.9	2.0 [1.0; 3.0]	<0.001
SU	10	0/10 (0.0%)	2/10 (20.0%)	6/10 (60.0%)	2/10 (20.0%)	0/10 (0.0%)	2.0 ± 0.7	2.0 [2.0; 2.0]	
DPP-4i	17	0/17 (0.0%)	3/17 (17.6%)	8/17 (47.1%)	5/17 (29.4%)	1/17 (5.9%)	2.2 ± 0.8	2.0 [2.0; 3.0]	
SGLT2i	25	0/25 (0.0%)	0/25 (0.0%)	5/25 (20.0%)	10/25 (40.0%)	10/25 (40.0%)	3.2 ± 0.8	3.0 [3.0; 4.0]	
GLP-1 RA	23	0/23 (0.0%)	0/23 (0.0%)	0/23 (0.0%)	11/23 (47.8%)	12/23 (52.2%)	3.5 ± 0.5	4.0 [3.0; 4.0]	
Insulin	45	0/45 (0.0%)	4/45 (8.9%)	29/45 (64.4%)	12/45 (26.7%)	0/45 (0.0%)	2.2 ± 0.6	2.0 [2.0; 3.0]	

Values are *n*/*N* (%) unless otherwise stated. The score ranges from 0 to 4 and assigns one point each for glycemic response, adiposity response, inflammatory response, and functional preservation. The global *p*-value refers to between-treatment differences in score distribution.

**Table 6:** Observed combinations of response domains.

Response Combination	Domains Present	*N*	%	Most Frequent Treatment Groups
GF	glycemic; functional preservation	51	30.7	Insulin 27; Metformin 8; DPP-4i 8
GIF	glycemic; inflammatory; functional preservation	27	16.3	Insulin 12; Metformin 6; DPP-4i 4
GAIF	glycemic; adiposity; inflammatory; functional preservation	25	15.1	GLP-1 RA 12; SGLT2i 10; Metformin 2
GAF	glycemic; adiposity; functional preservation	16	9.6	GLP-1 RA 8; Metformin 4; SGLT2i 4
F	functional preservation	10	6.0	Metformin 8; SU 1; DPP-4i 1
G	glycemic	7	4.2	Insulin 4; Metformin 1; SU 1
IF	inflammatory; functional preservation	6	3.6	Metformin 5; SU 1
GAI	glycemic; adiposity; inflammatory	6	3.6	SGLT2i 3; GLP-1 RA 3
AIF	adiposity; inflammatory; functional preservation	4	2.4	Metformin 3; DPP-4i 1
AF	adiposity; functional preservation	4	2.4	Metformin 4
GI	glycemic; inflammatory	3	1.8	Insulin 2; Metformin 1
None	none	2	1.2	Metformin 2
I	inflammatory	2	1.2	Metformin 1; DPP-4i 1
GA	glycemic; adiposity	2	1.2	SGLT2i 2
A	adiposity	1	0.6	Metformin 1

G = glycemic response; A = adiposity response; I = inflammatory response; F = functional preservation. Values are ordered by frequency. Domain combinations illustrate concordant and discordant multidimensional response patterns at the patient level.

**Table 7:** Exploratory univariable predictors of high multidimensional response.

Predictor	Contrast/Unit	OR	95% CI	*p*-Value	*N*
Age	per 1 SD higher	0.94	0.69–1.28	0.689	166
Diabetes duration	per 1 SD higher	1.19	0.88–1.62	0.259	166
Baseline HbA1c	per 1 SD higher	1.10	0.81–1.50	0.538	166
Baseline BMI	per 1 SD higher	0.83	0.61–1.13	0.248	166
Baseline fat mass %	per 1 SD higher	1.12	0.83–1.53	0.458	166
Baseline ln(hs-CRP)	per 1 SD higher	0.71	0.52–0.98	0.036	166
Baseline handgrip	per 1 SD higher	0.95	0.70–1.28	0.718	166
Medication adherence %	per 1 SD higher	1.28	0.94–1.75	0.118	166
Male sex	male vs. female	0.86	0.47–1.60	0.751	166
GLP-1 RA therapy	GLP-1 RA vs. all others	74.95	4.46–1258.90	<0.001	166
SGLT2i therapy	SGLT2i vs. all others	5.32	1.96–14.44	<0.001	166
Insulin therapy	insulin vs. all others	0.31	0.15–0.65	0.002	166
Resistance training	yes vs. no	0.96	0.47–1.96	1.000	166

Outcome: high multidimensional response, defined as score ≥ 3. Continuous variables are reported per 1 SD higher. OR, odds ratio; CI, confidence interval. Treatment variables compare each specified treatment class with all other treatment classes combined. The results are exploratory and should not be interpreted as causal estimates.

## Data Availability

The data that support the findings of this study are available from the corresponding author upon reasonable request.

## Abbreviations

A	Adiposity response
ACE	Angiotensin-converting enzyme
AF	Adiposity response and functional preservation
AIF	Adiposity response, inflammatory response, and functional preservation
BMI	Body mass index
CC BY	Creative Commons Attribution license
CI	Confidence interval
DPP-4	Dipeptidyl peptidase-4
DPP-4i	Dipeptidyl peptidase-4 inhibitor
eGFR	Estimated glomerular filtration rate
F	Functional preservation
G	Glycemic response
GA	Glycemic response and adiposity response
GAF	Glycemic response, adiposity response, and functional preservation
GAI	Glycemic response, adiposity response, and inflammatory response
GAIF	Glycemic, adiposity, inflammatory responses, and functional preservation
GF	Glycemic response and functional preservation
GI	Glycemic response and inflammatory response
GIF	Glycemic response, inflammatory response, and functional preservation
GIP	Glucose-dependent insulinotropic polypeptide
GLP-1	Glucagon-like peptide-1
GLP-1 RA	Glucagon-like peptide-1 receptor agonist
HbA1c	Glycated hemoglobin/hemoglobin A1c
HOMA-IR	Homeostatic model assessment of insulin resistance
hs-CRP	High-sensitivity C-reactive protein
I	Inflammatory response
IF	Inflammatory response and functional preservation
IL-6	Interleukin-6
IQR	Interquartile range
ln	Natural logarithm
MRS	Multidimensional response score
OR	Odds ratio
SD	Standard deviation
SGLT2	Sodium-glucose cotransporter-2
SGLT2i	Sodium-glucose cotransporter-2 inhibitor
SU	Sulfonylurea
T2DM	Type 2 diabetes mellitus
TNF-alpha	Tumor necrosis factor-alpha
